# The Classification and Prediction of Ferroptosis-Related Genes in ALS: A Pilot Study

**DOI:** 10.3389/fgene.2022.919188

**Published:** 2022-07-08

**Authors:** Qianqian Zhang, Huihui Zhao, Maotao Luo, Xi Cheng, Yanan Li, Qingyang Li, Zheng Wang, Qi Niu

**Affiliations:** Deartment of Geriatrics, The First Affiliated Hospital of Nanjing Medical University, Nanjing Medical University, Nanjing, China

**Keywords:** amyotrophic lateral sclerosis, ferroptosis and ferroptosis-related genes, WGCNA, LASSO, CHMP5, SLC38A1

## Abstract

Amyotrophic lateral sclerosis (ALS) is a neurodegenerative disease characterized by progressive muscle paralysis, which is followed by degeneration of motor neurons in the motor cortex of the brainstem and spinal cord. The etiology of sporadic ALS (sALS) is still unknown, limiting the exploration of potential treatments. Ferroptosis is a new form of cell death and is reported to be closely associated with Alzheimer’s disease (AD), Parkinson’s disease (PD), and ALS. In this study, we used datasets (autopsy data and blood data) from Gene Expression Omnibus (GEO) to explore the role of ferroptosis and ferroptosis-related gene (FRG) alterations in ALS. Gene set enrichment analysis (GSEA) found that the activated ferroptosis pathway displayed a higher enrichment score, and the expression of 26 ferroptosis genes showed obvious group differences between ALS and controls. Using weighted gene correlation network analysis (WGCNA), we identified FRGs associated with ALS, of which the Gene Ontology (GO) analysis displayed that the biological process of oxidative stress was the most to be involved in. KEGG pathway analysis revealed that the FRGs were enriched not only in ferroptosis pathways but also in autophagy, FoxO, and mTOR signaling pathways. Twenty-one FRGs (NR4A1, CYBB, DRD4, SETD1B, LAMP2, ACSL4, MYB, PROM2, CHMP5, ULK1, AKR1C2, TGFBR1, TMBIM4, MLLT1, PSAT1, HIF1A, LINC00336, AMN, SLC38A1, CISD1, and GABARAPL2) in the autopsy data and 16 FRGs (NR4A1, DRD4, SETD1B, MYB, PROM2, CHMP5, ULK1, AKR1C2, TGFBR1, TMBIM4, MLLT1, HIF1A, LINC00336, IL33, SLC38A1, and CISD1) in the blood data were identified as target genes by least absolute shrinkage and selection operator analysis (LASSO), in which gene signature could differentiate ALS patients from controls. Finally, the higher the expression of CHMP5 and SLC38A1 in whole blood, the shorter the lifespan of ALS patients will be. In summary, our study presents potential biomarkers for the diagnosis and prognosis of ALS.

## Introduction

Amyotrophic lateral sclerosis (ALS) is a fatal and incurable neurodegenerative disease affecting the motor nervous system in the brain and spinal cord, which is the most common subtype of motor neuron disease (MND). Worldwide, ALS is a severe public health problem with an incidence of 1.75–4.42 in 100,000 and the median survival time is only 2–4 years (Chiò et al., 2014; De Marchi et al., 2019; Xu et al., 2020). About 10% of the ALS cases are classified as familial ALS (fALS) caused by genetic mutations and the remaining 90% of cases are termed sporadic ALS (sALS) (Ajroud-Driss and Siddique, 2015). Riluzole and edaravone are the two approved treatments for ALS; however, they only have a mild effect (Bordoni et al., 2020). Despite a lot of pathologic mechanisms having been proposed, the etiology of sALS is still unknown; moreover, most sALS cases are diagnosed by clinical symptoms and signs without definitive diagnostic tests, which significantly hampers the development of potentially effective drugs.

In 2012, a novel form of cell death was discovered, which is different from typical programmed cell death processes (necrosis, autophagic, and apoptosis), characterized by excessive iron-dependent lipid peroxidation and named ferroptosis (Dixon et al., 2012). Ferroptosis is closely associated with the iron metabolism and has been reported to be related to several diseases, such as cancer (Yu et al., 2017), stroke (Alim et al., 2019), myocardial infarction (Park et al., 2019), and a variety of neurodegenerative diseases, such as Alzheimer’s disease (AD) (Stockwell et al., 2017), Parkinson’s disease (PD) (Do et al., 2016; Masaldan et al., 2019), and ALS (Wang et al., 2021). In induced pluripotent stem cells induced by patients with sporadic ALS, lipid peroxidation and ferroptosis played important roles in motor neuron cell death (Fujimori et al., 2018). In the transgenic mouse model of ALS, using iron chelators could significantly reduce the iron level and increase the mean life span (Moreau et al., 2018; Wang et al., 2011). The specific pathogenesis of ferroptosis in ALS remains unclear. Previous neuroimaging studies have found the deposition of iron in the involved brain and spinal regions in ALS (Andersen et al., 2014). Human postmortem research demonstrates that ferroptosis, but not necroptosis, could be more important in the mediation of motor neuron death (Wang et al., 2021). In addition, as a key antioxidant enzyme in suppressing ferroptosis, the glutathione peroxidase 4 (GPX4) expression level is significantly reduced in both fALS and sALS (Wang et al., 2021). In the transgenic mouse model of ALS (SOD1^G93A^, TDP-43, and C9orf72), conditional ablation of GPX4 leads to obvious degeneration of motor neurons and significantly shortens the survival time. Contrastingly, the overexpression of GPX4 exhibits delayed disease onset and improved motor function (Chen et al., 2015, 2021; Evans et al., 2022; Wang et al., 2021). Prior research has confirmed that the mechanism of ferroptosis is very complex and can be modulated by numerous genes (Zhou and Bao, 2020). However, whether these ferroptosis-related genes (FRGs) have a potential prognostic and predictive role in ALS remains largely unknown.

Here, publicly available mRNA expression data of ALS postmortem human specimens were first used to perform the cluster and differential expression analysis to identify the FRG target genes, which were then utilized to construct a predictive multigene signature and validated in the whole blood chip-seq data of ALS. Finally, we explored the survival of the FRG target genes in the blood data to evaluate the prognostic gene signature for ALS.

## Materials and Methods

### Data Source

**FIGURE 1:**
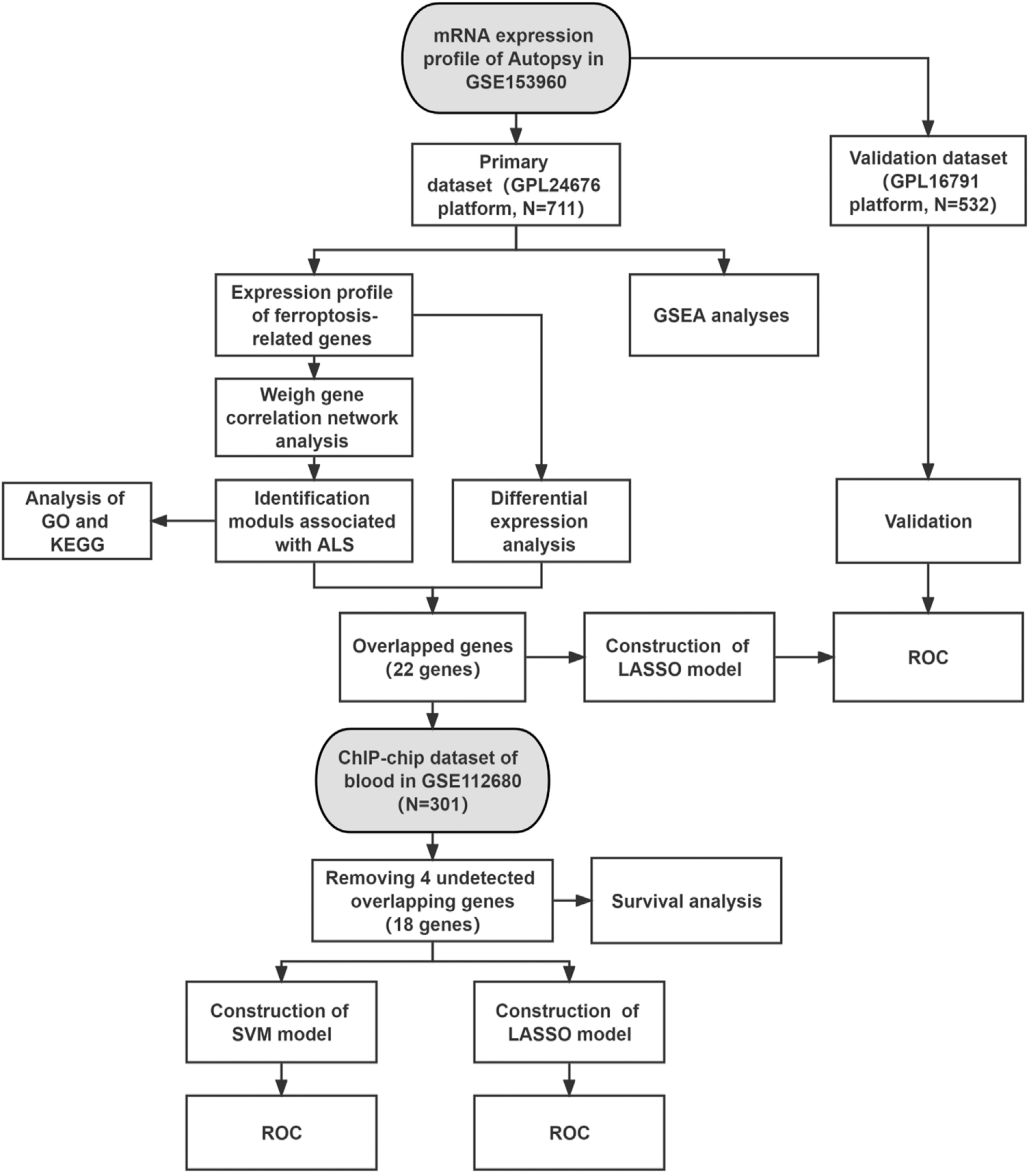
Workflow of this study. Abbreviations: ALS, amyotrophic lateral sclerosis; GSEA, gene set enrichment analysis; KEGG, Kyoto encyclopedia of genes and genomes; GO, gene ontology; LASSO, least absolute shrinkage and selection operator; SVM, support vector machine; ROC, receiver operating characteristic.

The two datasets in this study were collected from the Gene Expression Omnibus (GEO) database (https://www.ncbi.nlm.nih.gov/geo/) (GSE153960 and GSE112680). The GSE153960 dataset contains mRNA expression data across the brain and spinal cord of postmortem human specimens from 1,838 samples in the New York Genome Center (NYGC) ALS Consortium, including non-neurological control, ALS spectrum, familial ALS, and other neurological disorders or motor neuron disease. Depending on the platform, the GSE153960 dataset consisted of two different parts: one was based on the GPL24676 and was selected as the primary dataset and the other one was based on the GPL16791 and was referred to as the secondary dataset. We chose the non-neurological controls and ALS patients in our analysis, which included 521 ALS patients and 190 non-neurological controls and 442 ALS patients and 90 non-neurological controls in the primary and secondary datasets, respectively. Finally, the GSE112680 dataset was used to search for prognostic and predictive novel biomarkers of ALS, which included 376 whole-blood samples of ChIP–chip data (control, ALS, and ALS-mimic) (Van Rheenen et al., 2018). After the removal of ALS-mimic, we obtained 164 ALS patients and 137 controls in the blood dataset (Figure 1).

### Data Processing

All data were processed and analyzed using R (version 4.1.0). For the GSE153960 dataset, the DESeq2 package (version 1.32.0) was used to normalize raw counts by the variance stabilizing transformation (vst) function and shrink the log2FoldChanges by the lfcShrink function (Love et al., 2014). A total of 348 Ferroptosis-related genes (FRGs) that drive, suppress or mark ferroptosis were downloaded from public FerrDb database after removing the duplicates (Zhou and Bao, 2020) (Supplementary Table S1). For the GSE112680 dataset, the normalization and log-transformed was performed by the limma package (version 3.48.3).

### Gene Set Enrichment Analysis (GSEA)

The GSEA analysis was processed by the gseKEGG function in the R package clusterProfiler (version 4.0.5) (nPerm = 10,00, minGSSize = 20, *p* = 0.05). The GSEA plots were generated by the R package enrichplot (Version 1.12.2) and ggpolt2 (version 3.3.5).

### Weighted Gene Correlation Network Analysis

Using the R WGCNA package (version 1.70-3; https://cran.r-project.org/package=WGCNA), we performed WGCNA analysis of the FRGs extracted from the primary dataset (Langfelder and Horvath, 2008). All processed expressions of data were computed to construct a similarity matrix by using Pearson’s correlation analysis. Subsequently, the integrated pickSoftThreshold function was used to calculate a suitable power of β to achieve a scale-free co-expression network. The minimum size of 30 was set to obtain co-expression modules in which the similar expression patterns of FRGs were clustered into different color modules. Finally, the ALS-related modules of FRGs were identified by Pearson’s correlation analysis of the calculation of phenotype and each module (Pei et al., 2020).

### Gene Function Annotation and Pathway Analysis

The genes of all the identified WGCNA ALS-related FRGs modules were performed to the Gene Ontology (GO) and Kyoto Encyclopedia of Genes and Genomes (KEGG) pathway analyses using the *clusterProfiler* R package (version 4.0.5) (Yu et al., 2012). For statistically significant differences, the cutoff was set to a false discovery rate (FDR) *p* < 0.05. In addition, the top 10 KEGG pathways were shown as bubble charts, while using the bar chart to display the top eight GO terms of biological process (BP), cellular component (CC), and molecular function (MF).

### Identification of Ferroptosis-Related Gene Target Genes

The DESeq2 R package was used for differential gene screening of FRGs that were extracted from the primary dataset. Considering the log2FoldChanges was shrank in the processing, we set the | log2 (fold change)| > 0.5 and adjusted *p* < 0.05 as the cut-off criteria (Gormally et al., 2014). The obtained FRG differential genes and the identified WGCNA ALS-related FRGs were crossed to obtain the overlapped genes, which were selected as the FRG target genes. Meanwhile, the expression correlation analysis of the overlapped genes was performed by the Pearson correlation coefficients.

### Construction of Least Absolute Shrinkage Selection Operator and Support Vector Machine

Using the R package glmnet (version 4.1-2) and e1071 (version 1.7–9), we constructed the LASSO (primary dataset; blood dataset) and SVM (blood dataset) models with the FRG target genes, respectively. The LASSO is a method of compression estimate with 5-fold cross-validation (family = binomial, measure type = deviance, lambda = the minimum value), which is used to simplify the model and to prevent overfitting. The ferroptosis-related score of the LASSO model was computed with the equation ferroptosis-related score = Σ expgenei* βi, where expgenei is the relative gene expression in the signature for patient i, and βi is the LASSO regression coefficient of gene i. SVM is a discriminative algorithm which has shown promising and better classification performance compared with other methods (Scholkopf et al., 1997). We selected the Radial Basis Function (RBF kernel) as the kernel function and performed 5-fold cross-validation to construct and validate the SVM model (type = eps-regression, the other parameters set by default). Moreover, the primary dataset and blood dataset were divided into the training (70%) and test (30%) sets that represented the training and internal validation cohort, respectively. Finally, the receiver operating characteristic (ROC) curve analyses were used to display the predicted efficacy of the occurrence of ALS in the LASSO model (training and validation cohort, respectively) and the SVM model (confusion matrices of training and validation) with the pROC package (version 1.18.0) (Robin et al., 2011). The overall accuracy of the model was evaluated using the area under the curve (AUC) (area under the ROC Curve) and a value of over 70% was considered acceptable (Rice and Harris, 2005).

### Survival Analysis

We performed the Kaplan–Meier survival analysis (log-rank) of the clinical characteristics (gender, onset age, and site) and the FRG target genes in the blood dataset using the R survival (version 3.2–11) and survminer packages (version 0.4.9). To detect the effect of onset age, age stratification was performed by 55 and 65 years, respectively. For the FRG target genes, we set the median value of expression levels as a cut-off score that over the median were classified as high expression and the others as low expression. *p*-values of less than 0.05 were deemed indicative of survival differences.

## Result

### GSEA

**FIGURE 2:**
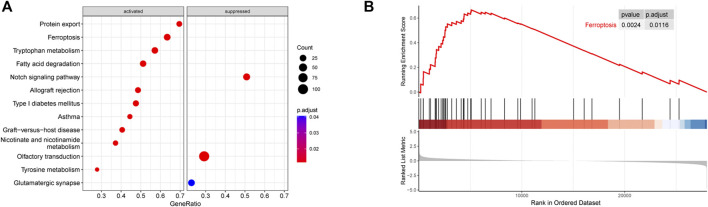
Results of GSEA (KEGG pathways). **(A)** Distribution of gene ratio for the top 10 signaling pathways. **(B)** Enrichment plot of the ferroptosis pathway. Abbreviations: GSEA, gene set enrichment analysis; KEGG, Kyoto encyclopedia of genes and genomes.

Figure 1 shows the whole flow diagram of this study, and Figure 2 is the result of GSEA using the KEGG pathway gene sets in the primary dataset. GSEA is a threshold-free approach that can identify significantly differentially expressed gene sets without being affected by the internal correlation difference (Subramanian et al., 2007). A total of 126 enriched pathways were upregulated and three were downregulated by GSEA (Supplementary Table S2). Figure 2A lists the top 10 enriched activated pathways and three suppressed pathways, including protein export, ferroptosis, tryptophan metabolism, notch signaling pathway, olfactory transduction, and glutamatergic synapse. Of these, the ferroptosis pathway was significantly enriched (adjusted *p* = 0.0116) (Figure 2B), and in which the 26 gene expression levels show striking differences between the control and ALS group in the primary datasets using Student’s t test (*p* < 0.05) (Supplementary Figure S1).

### WGCNA

**FIGURE 3:**
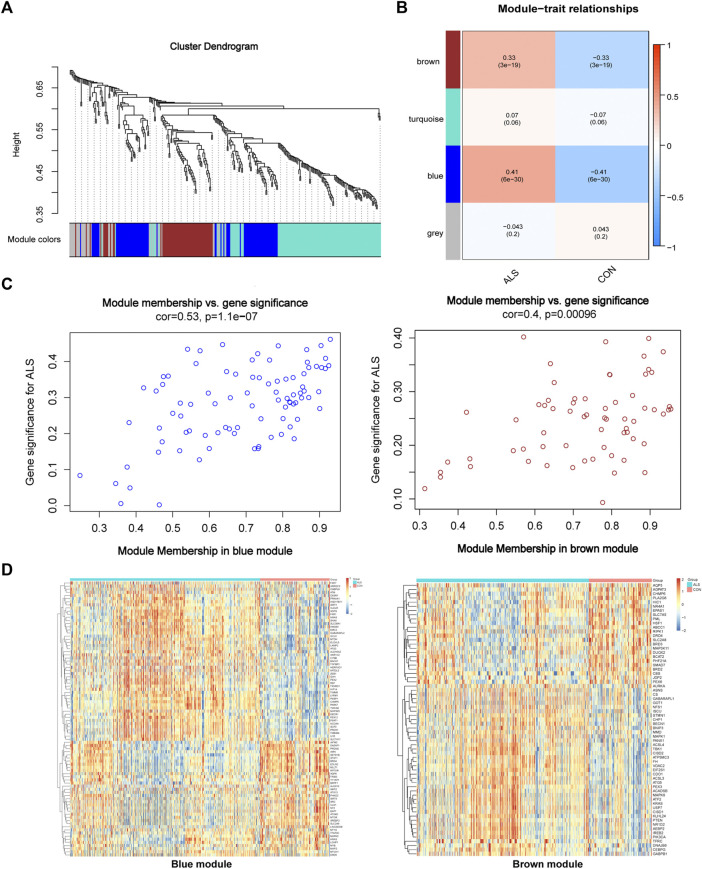
Visualization of WGCNA results. **(A)** WGCNA cluster dendrogram groups genes into distinct four modules. **(B)** Correlation between each module’s eigengene and phenotype. Two modules were positively correlated with ALS, namely, the brown and blue modules. All modules are marked with different colors. **(C)** Scatter plot of module eigengenes in the brown and blue modules that significantly correlated with ALS (*p* < 0.05). **(D)** Heatmap profiling of the genes of brown and blue modules derived from WGCNA. Abbreviations: WGCNA, weighted gene correlation network analysis; ALS, amyotrophic lateral sclerosis.

We constructed a gene co-expression network with the expression profiles of FRGs in the primary dataset (Figure 3) and calculated a suitable β to build a scale-free network (β = 1, scale-free R^2^ > 0.85) (Guo et al., 2021). A total of 4 modules were identified and labeled with different colors (Figure 3A), in which the blue (cor = 0.41, p = 6e-30) and brown modules (cor = 0.33, p = 3e-19) were positively correlated with ALS (Figure 3B). Next, the correlation of each gene with phenotype was analyzed, and the two modules represented a significant association between gene-trait significance and module membership (blue: cor = 0.53, *p* = 1.1e-07; brown: cor = 0.4, *p* = 0.00096) (Figure 3C). Figure 3D shows the heatmap of the correlations between genes and clinical phenotypes in the two modules.

### Gene Ontology and Kyoto Encyclopedia of Genes and Genomes Pathway Analyses

**FIGURE 4:**
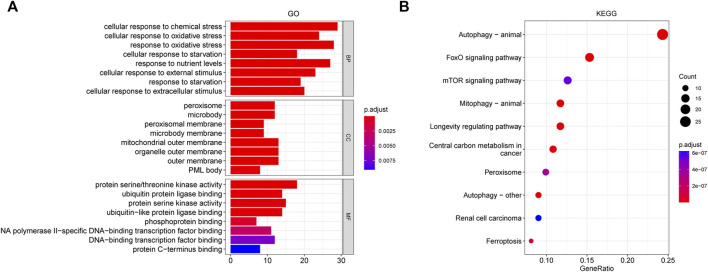
GO and KEGG analysis for the whole ALS- related genes of brown and blue modules derived from WGCNA. **(A)** GO pathway analysis, including BP, CC, and MF. **(B)** Top 10 results of KEGG pathway enrichment analysis. The color represents the adjusted *p* value, and the size of the dots and bar represents the number of the genes. Abbreviations: GO, gene ontology; KEGG, Kyoto encyclopedia of genes and genomes; WGCNA, weighted gene correlation network analysis; BP, biological process; CC, cellular component; MF, molecular function.

A total of 153 genes were obtained from the WGCNA identified blue and brown modules that were used to perform GO and KEGG pathway analyses (Figure 4). There were several biological processes that were enriched, including cellular response to chemical or oxidative stress, and external or extracellular stimulus. In the enrichment of molecular function, the activity of protein serine/threonine kinase, protein serine kinase, and ubiquitin−like protein ligase or phosphoprotein binding was obviously involved. In the cellular component category, peroxisome, mitochondrial outer membrane, organelle outer membrane, and outer membrane were the main enriched terms (Figure 4A). (Supplementary Table S3). Figure 4B showed the top 10 results of the KEGG pathway enrichment analysis, which mainly involved autophagy, FoxO signaling, mTOR signaling pathway, and ferroptosis (Supplementary Table S4).

### Ferroptosis-Related Genes Target Genes

**FIGURE 5:**
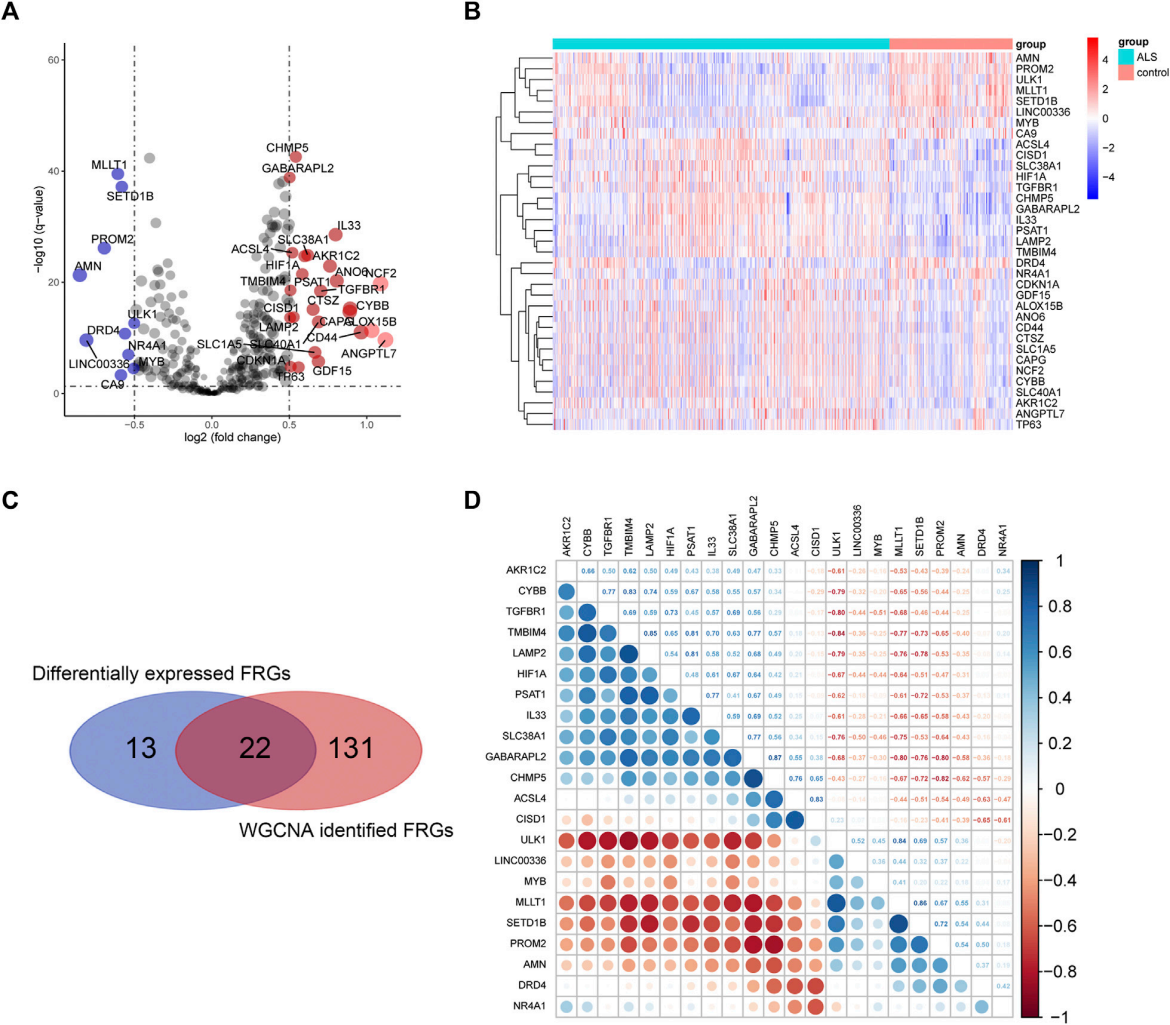
Overlapping differentially expressed FRGs and ALS-related FRGs derived from WGCNA. **(A)** and **(B)** represent the volcano plot and heat map of differential FRG expression analysis. **(C)** Twenty-two overlapping genes were identified between differentially expressed FRGs and ALS-related FRGs derived from WGCNA. **(D)** Heatmap of Pearson’s correlation across 22 overlapping genes. Abbreviations: ALS, amyotrophic lateral sclerosis; WGCNA, weighted gene correlation network analysis; FRGs, ferroptosis-related genes.

A total of 35 differentially expressed genes (DEGs) between ALS and control group were obtained from the FRGs that extracted from processed primary dataset using the cut-off criteria of | log2 (fold change)| > 0.5 and adjusted *p* < 0.05 (Figure 5A, Figure 5B). Then, intersecting the 35 DEGs-FRGs and the 153 WGCNA identified ALS-related FRGs resulted in 22 FRG target genes (Figure 5C). Moreover, we performed Pearson correlation coefficient analysis to calculate the correlations among the overlapped 22 FRG expression levels (Figure 5D), and the result showed that all 22 genes were correlated with each other. Among them, CYBB and TMBIM4 (correlation = 0.83), TMBIM4 and LAMP2 (correlation = 0.85), LAMP2 and PSAT1 (correlation = 0.81), GABARAPL2 and CHMP5 (correlation = 0.87) were the four pairs of genes that showed the most positive correlation. However, TGFBR1 and ULK1 (correlation = -0.80), TMBIM4 and ULK1 (correlation = -0.84), GABARAPL2 and MLLT1 (correlation = -0.80), CHMP5 and PROM2 (correlation = -0.82), were the four pairs of most negatively correlated genes.

### Construction of the Least Absolute Shrinkage and Selection Operator Model in the Autopsy Data

**FIGURE 6:**
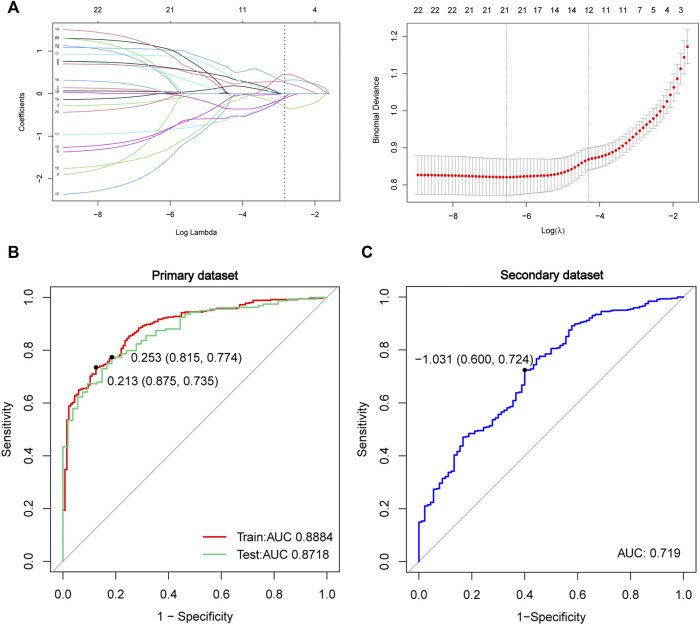
Construction and validation of the LASSO model in the primary data. **(A)** Construction of the LASSO model by the overlapped 22 genes. The ROC curve in the primary dataset of training data (red lines, N = 498, AUC = 0.8884), testing data (green lines, N = 213, AUC = 0.8718) **(B)**, and in the secondary dataset (blue lines, N = 532, AUC = 0.719) **(C)** Red line represents training data, the green line represents test data, and the blue line represents the secondary dataset. Abbreviations: LASSO, least absolute shrinkage and selection operator; ROC, receiver of characteristic curve; AUC, area under curve.

The LASSO analysis was employed to further screen core genes of the overlapped 22 FRGs and construct the prediction model in the primary dataset (Figure 6A). In our study, there were 21 genes with non-zero coefficients that were selected in the model: NR4A1 expression ∗0.05013 + CYBB expression ∗0.06870 + DRD4 expression ∗(-0.18833) + SETD1B expression ∗ 1.04753562 + LAMP2 expression ∗0.56448 + ACSL4 expression ∗(−1.03872649) + MYB expression ∗0.66426 + PROM2 expression ∗0.63262 + CHMP5 expression ∗(−1.05494) + ULK1 expression ∗(−1.84926) + AKR1C2 expression ∗(−0.88291) + TGFBR1 expression ∗(−0.98841) + TMBIM4 expression ∗1.13877 + MLLT1 expression ∗1.13996 + PSAT1 expression ∗(−1.45143) + HIF1A expression ∗0.12640 + LINC00336 expression ∗0.89801 + AMN expression ∗(−0.02644) + SLC38A1 expression ∗(−0.23000) + CISD1 expression ∗0.73836 + GABARAPL2 expression ∗0.63893. Next, ROC curves were used to measure the accuracy of the predictive model in the primary dataset (train and test) and also used for external validation in the secondary dataset (Figure 6B). The AUC was 0.8884 (training set, red line, sensitivity = 0.875, specificity = 0.735), 0.8718 (test set, green line, sensitivity = 0.815, specificity = 0.774), and 0.719 (secondary dataset, blue line, sensitivity = 0.600, specificity = 0.724), respectively. This suggests that these 21 gene signatures could be used as a diagnostic biomarker for ALS.

### Analysis of Diagnostic Performance and Kaplan-Meier in the Blood Data

**FIGURE 7:**
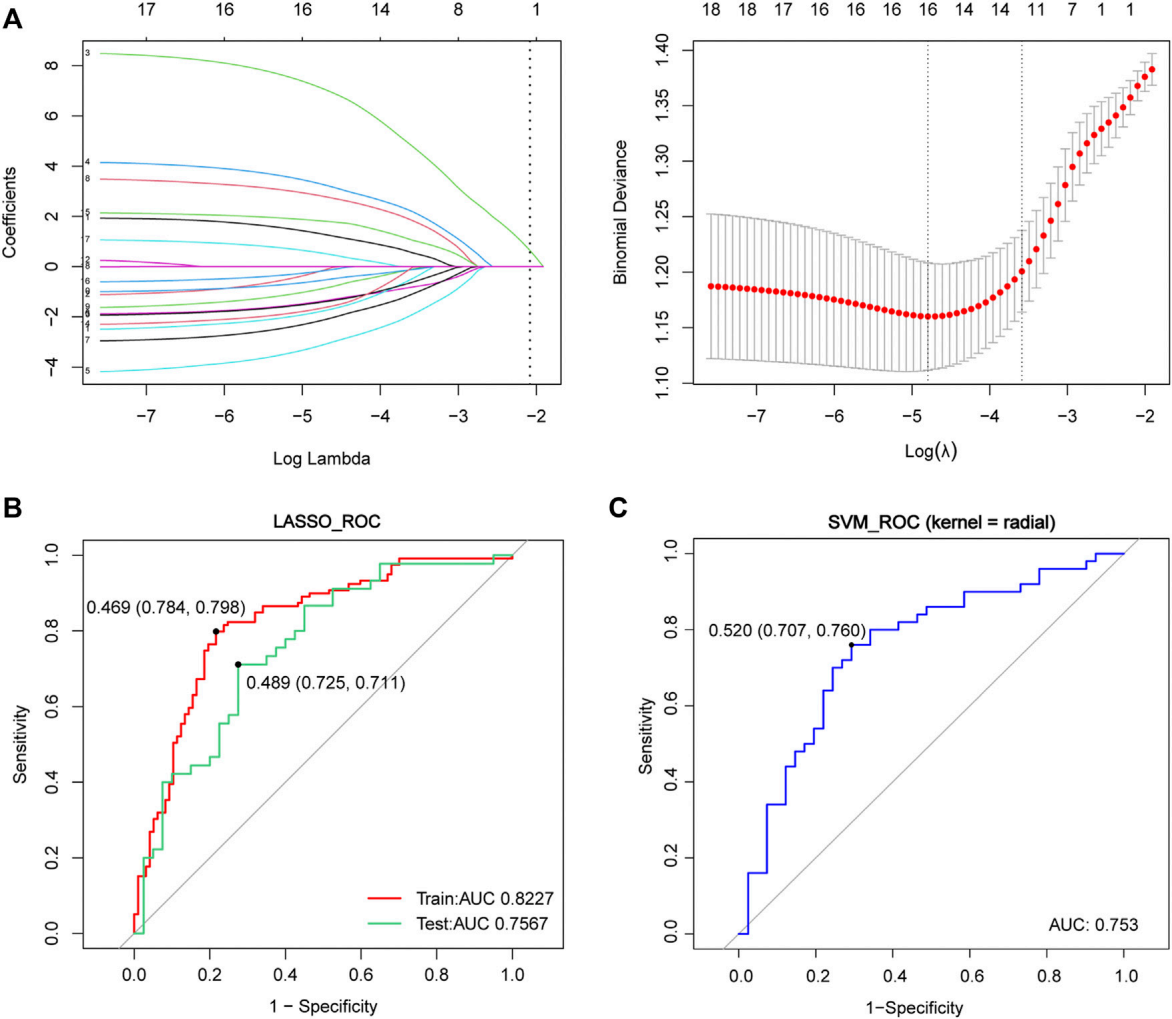
Construction of the LASSO and SVM model in the whole blood data. **(A)** Construction of the LASSO model. **(B)** ROC curve in the blood dataset of training data (red lines, N = 216, AUC = 0.8227), testing data (green lines, N = 85, AUC = 0.7567) and in the SVM model (blue lines, N = 301, AUC = 0.753) **(C)** Red and green lines represent the ROC of training and test data in the LASSO model, respectively, where the blue line represents the ROC in the SVM model. Abbreviations: LASSO, least absolute shrinkage and selection operator; ROC, receiver of characteristic curve; AUC, area under curve; SVM, support vector machine.

To further explore the diagnostic performance of the FRGs target genes in the whole blood, we adopted two approaches to construct the predictive models (Figure 7). A total of 18 FRG target genes were involved in the two models because of 4 genes undetected in the blood data (CYBB, LAMP2, ACSL4, and AMN). In the LASSO model, the AUC was 0.8227 in the training set (red line, sensitivity = 0.784, specificity = 0.798) and 0.7567 in the test set (green line, sensitivity = 0.725, specificity = 0.711) (Figure 7A and Figure 7B), which contained 16 genes with non-zero coefficients: NR4A1 expression ∗1.32357 + DRD4 expression ∗(−0.20650) + SETD1B expression ∗ 7.15886 + MYB expression ∗3.31530 + PROM2 expression ∗(−3.17261) + CHMP5 expression ∗(−1.39924) + ULK1 expression ∗(−2.17756) + AKR1C2 expression ∗2.83465 + TGFBR1 expression ∗(−0.91465) + TMBIM4 expression ∗(−0.56885) + MLLT1 expression ∗(−1.80802) + HIF1A expression ∗(−1.43793) + LINC00336 expression ∗(−1.68296) + IL33 expression ∗1.82787 + SLC38A1 expression ∗(−0.19496) + CISD1 expression ∗0.55844. Moreover, the ROC curves of the SVM model displayed the results for the confusion matrices of training and validation using the 18 FRGs (blue line, AUC = 0.768, sensitivity = 0.707, and specificity = 0.780). These results implied that the FRG target gene signatures in blood also had the diagnostic potential for ALS.

**FIGURE 8:**
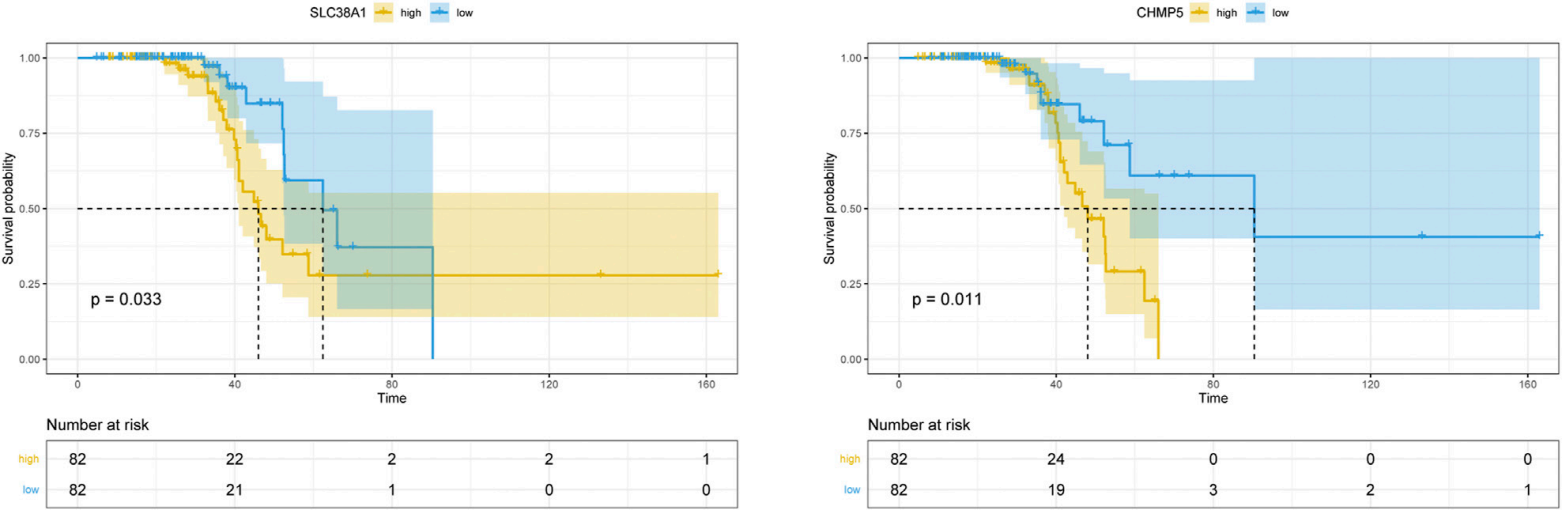
Kaplan–Meier survival curve of the 18 overlapping FRGs in blood data. CHMP5 and SLC38A1 revealed significant survival differences (*p* < 0.05).

Finally, Kaplan-Meier analyses were performed to evaluate the survival for ALS of the FRG target genes and clinical characteristics in blood data. In our study, gender, onset site (bulbar or spinal), and onset age didn’t show an influence on survival (Supplementary Figure S2). Figure 8 displayed the result of the FRG target gene survival analyses, which showed that the higher expression of CHMP5 (*p* = 0.011) and SLC38A1 (*p* = 0.033) were significantly correlated with worse survival outcome.

## Discussion

ALS is now widely considered as a multifactorial and highly heterogeneous disease. The lack of understanding of the pathogenesis of ALS and specific biomarkers has seriously hampered the development of clinically effective drugs. In our study, we systematically investigated the expression and constructed predictive models with the FRG target genes in the autopsy (22 genes) and blood samples (18 genes) of ALS, which showed strong predictive ability and successfully passed verification. Moreover, functional analyses showed that autophagy, mTOR signaling, and ferroptosis pathways were enriched. Finally, we found that the high expression of CHMP5 and SLC38A1 was closely related to the ALS patient’s prognosis, which showed a shorter lifespan.

GSEA has been widely used to perform gene set enrichment testing, which is based on enriched molecular signatures instead of differential gene expression (Subramanian et al., 2005). In the top 10 pathways that were enriched using KEGG pathway gene sets, the activated ferroptosis pathway displayed a higher enrichment score and the expression of 26 ferroptosis genes showed obvious group differences, which supported the hypothesis of ferroptosis being involved in the pathogenesis of ALS (Wang et al., 2021). In physiological situations, iron is essential for life activities and is involved in lots of neuronal functions. Previous research has reported iron metabolism deregulation in ALS patients and mouse models (Ignjatović et al., 2013; Jeong et al., 2009; Wang et al., 2020). In clinical studies, increased iron content (serum ferritin) is associated with a faster deterioration and shorter survival time in ALS (Nadjar et al., 2012; Paydarnia et al., 2021; Sun et al., 2019; Yu et al., 2018), while in the transgenic mouse model of ALS, using iron chelators could significantly reduce the iron level and increase the mean life span (Wang et al., 2011; Moreau et al., 2018). Moreover, the overexpression of GPX4 (a critical enzyme in suppressing ferroptosis) could improve both motor function and the prognosis of ALS mice, which provides a potential therapeutic target for ALS.

Using WGCNA, we identified 153 FRGs that were positively correlated with ALS from two modules, which were then subjected to the KEGG and GO analyses. The GO analysis displayed that biological processes of oxidative stress were the most involved, which was in accordance with the definition of ferroptosis. As reported in previous studies, iron could pass through the blood-brain barrier to enter the brain by transferrin-mediated iron transcytosis (Ke and Qian, 2007; Song et al., 2018), which leads to iron overload that induces oxidative stress and neuronal death (Jellinger, 1999). In addition, as a classical antioxidant, edaravone could effectively attenuate oxidative stress (Shimazaki et al., 2010) and prevent ferroptosis in ALS (Homma et al., 2019). Concerning molecular function, serine/threonine kinases were the most implicated in the ALS-associated FRGs. It is well known that, more than 25% of human protein kinases belong to serine/threonine protein kinases and are involved in regulating numerous signaling transduction cascades (Rask-Andersen et al., 2014), including the Mitogen-activated protein kinase (MAPK) family, Akt kinase (protein kinase B), Mammalian target of rapamycin (mTOR), and so on, which are reported to be strongly linked with the development of ALS (Halon-Golabek et al., 2019; Sama et al., 2017; Saxena et al., 2013). Notably, in the KEGG pathway analysis, we found the autophagy, ferroptosis, FoxO, and mTOR signaling pathways were activated. Numerous studies have reported the compromised autophagy in ALS (Chua et al., 2021; Evans and Holzbaur, 2019), which is negatively regulated by the mTOR pathway and induced by Foxo (Juhász et al., 2007; Yuan et al., 2015). Currently, the crosstalk between autophagy and ferroptosis is not well understood. Recent research concluded that autophagy could promote ferroptosis by degrading ferritin (Hou et al., 2016), and ferroptosis may be an autophagic cell death process (Gao et al., 2016). Together, there is a close association between autophagy and ferroptosis. Further exploration of the mechanism of the crosstalk is important for finding new therapeutic targets.

Considering the tissue-specificity, spatial-specificity and temporal-specificity of gene expression, we constructed different models in the autopsy and blood samples, respectively. 21 FRGs (NR4A1, CYBB, DRD4, SETD1B, LAMP2, ACSL4, MYB, PROM2, CHMP5, ULK1, AKR1C2, TGFBR1, TMBIM4, MLLT1, PSAT1, HIF1A, LINC00336, AMN, SLC38A1, CISD1, and GABARAPL2) in the primary data and 16 FRGs in the blood data (NR4A1, DRD4, SETD1B, MYB, PROM2, CHMP5, ULK1, AKR1C2, TGFBR1, TMBIM4, MLLT1, HIF1A, LINC00336, IL33, SLC38A1, and CISD1) were identified as target genes by using LASSO analysis, which signature showed better prediction ability in distinguishing ALS from controls. CYBB (also called NOX2), catalyzes the formation of ROS and was thought to be a new drug target and biomarker in neurodegenerative diseases (Sorce et al., 2017). In ALS patients, people with low NOX2 activity live longer (1-year increase in survival) (Sorce et al., 2017). In the ALS SOD1 transgenic mouse, the inactivation of NOX2 delays neurodegeneration and extends survival (Sorce et al., 2017). ULK1 is an autophagy inducing kinase that is reported to be more hyperactive in ALS mouse and could be regulated by C9orf72 (one of the well-known genetic causes of ALS) (Bandyopadhyay et al., 2014; Webster et al., 2016). HIF1A is a key transcription factor in maintaining oxygen homeostasis and is involved in the motor neuron degeneration of ALS (Nomura et al., 2019). Moreau et al. (Moreau et al., 2011) reported that the HIF-1 pathway of sporadic ALS patients showed significant abnormalities during hypoxia and could be a novel target for ALS therapy. In a recent study, the investigators found the loss of TGFBR1 in SOD1 mice, which suppressed the phagocytosis of microglia (Butovsky et al., 2015). LAMP2, a heavily glycosylated protein, correlated closely with inflammatory and lysosomal accumulation in the central nervous system (Rothaug et al., 2015). Chen et al. (Cheng et al., 2018) found that progressive lysosomal deficits existed in the familial amyotrophic lateral sclerosis-linked SOD1^G93A^ mouse. However, they didn’t find evidence to support the LAMP1/2 as the specific markers to assess lysosome distribution in neurodegenerative diseases, which requires additional studies to confirm in the future. Notably, although these genes have not been demonstrated to be directly associated with ALS in previous studies, NR4A1 (Zhao et al., 2018) and DRD4 (Lin et al., 2012) are reported in AD, ACSL4 seems to be a biomarker of ferroptosis (Yuan et al., 2016), and SETD1B is associated with syndromic neurodevelopmental disorder (Weerts et al., 2021). Moreover, using KM analysis, we didn’t find the survival differences in the age of onset, gender, and onset site, which might be due to the method of selected stratification approach and the inclusion of more long-surviving patients (Chiò et al., 2009; Magnus et al., 2002). Importantly, we found that the higher the expression level of CHMP5 and SLC38A1, the worse the prognosis. CHMP5 has not been reported in previous ALS related research, and SLC38A1 is a main transporter of glutamine and has been reported as a positive regulator of mTOR complex 1 (mTORC1), which is closely connected with autophagy and plays an essential role in many neurodegenerative diseases (Lipton and Sahin, 2014; Yamada et al., 2019). Overall, these results might offer a new point in the exploration of molecular mechanisms and the seeking of prognostic biomarkers of ALS.

There were also some limitations to be mentioned. First, because of the four genes undetected in the whole-blood samples of ChIP–chip data, we only used 18 FRGs to perform LASSO analysis, which may miss some important contributions to the blood model construction. Second, there were no external validation blood datasets in this study; however, we used two different methods that had 5-fold cross-validation and both showed good predictive ability for ALS. Third, the ALS gene signature analysis was based on public data, the findings here need future experimental and clinical validation.

## Conclusion

The results of this study prove that ferroptosis-related genes are closely related to ALS. The identified 21 FRGs and 16 FRGs can differentiate ALS patients from controls in autopsy and blood samples, respectively, which are potential biomarkers for disease diagnosis. Finally, higher expression of CHMP5 and SLC38A1 is related to shorter survival, which may be potential biomarkers for the prognosis of ALS.

## Data Availability

Publicly available datasets were analyzed in this study. The names of the repository/repositories and accession number(s) can be found in the article/Supplementary Material.
