# Supplementary figures and images for: The Classification and Prediction of Ferroptosis-Related Genes in ALS: A Pilot Study
Source: Front Genet. 2022 Jul 8;13:919188. doi: 10.3389/fgene.2022.919188 (PMC9305067; doi:10.3389/fgene.2022.919188)

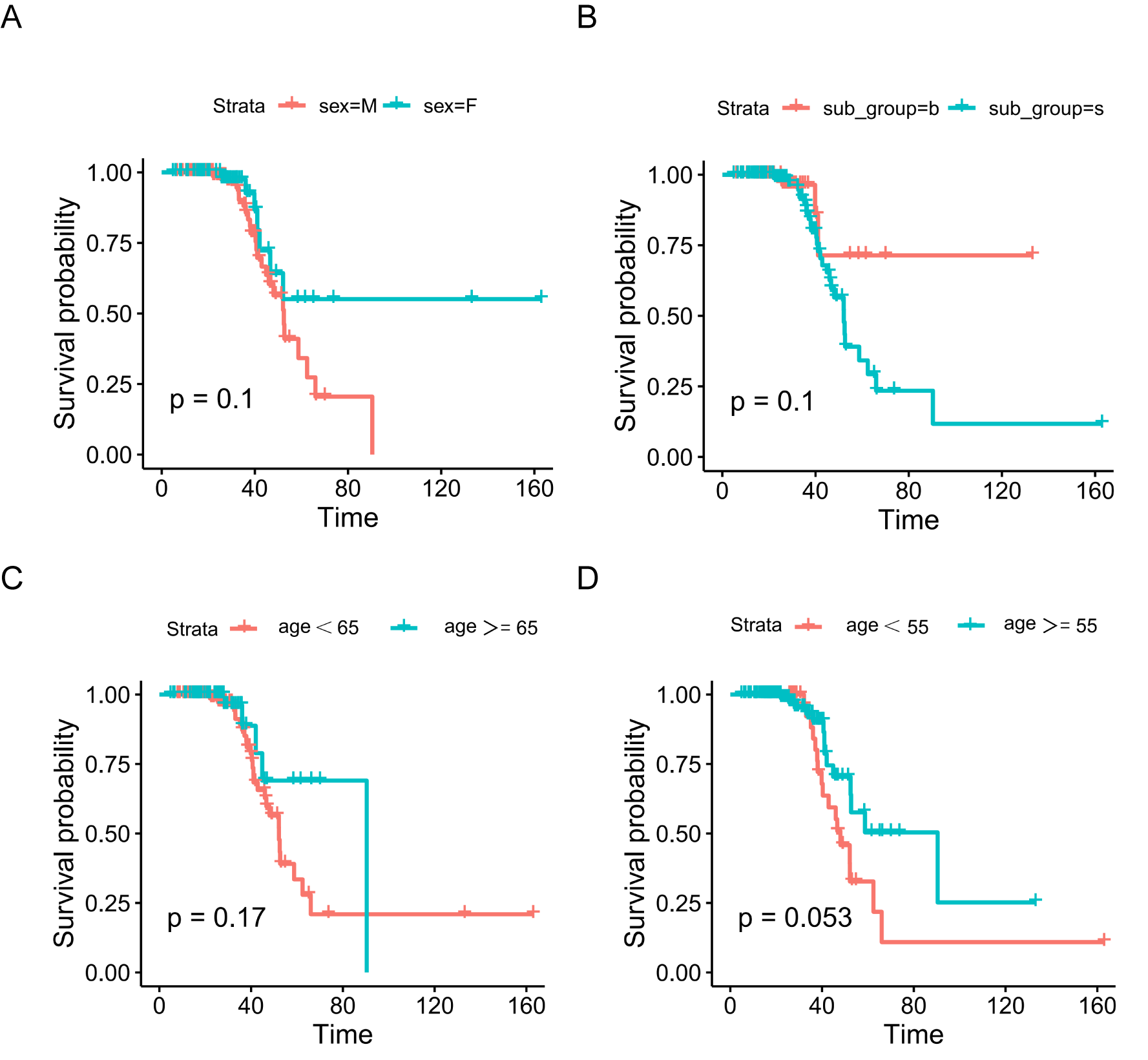

Supplement: Supplementary file 3 [file Image2.TIF]

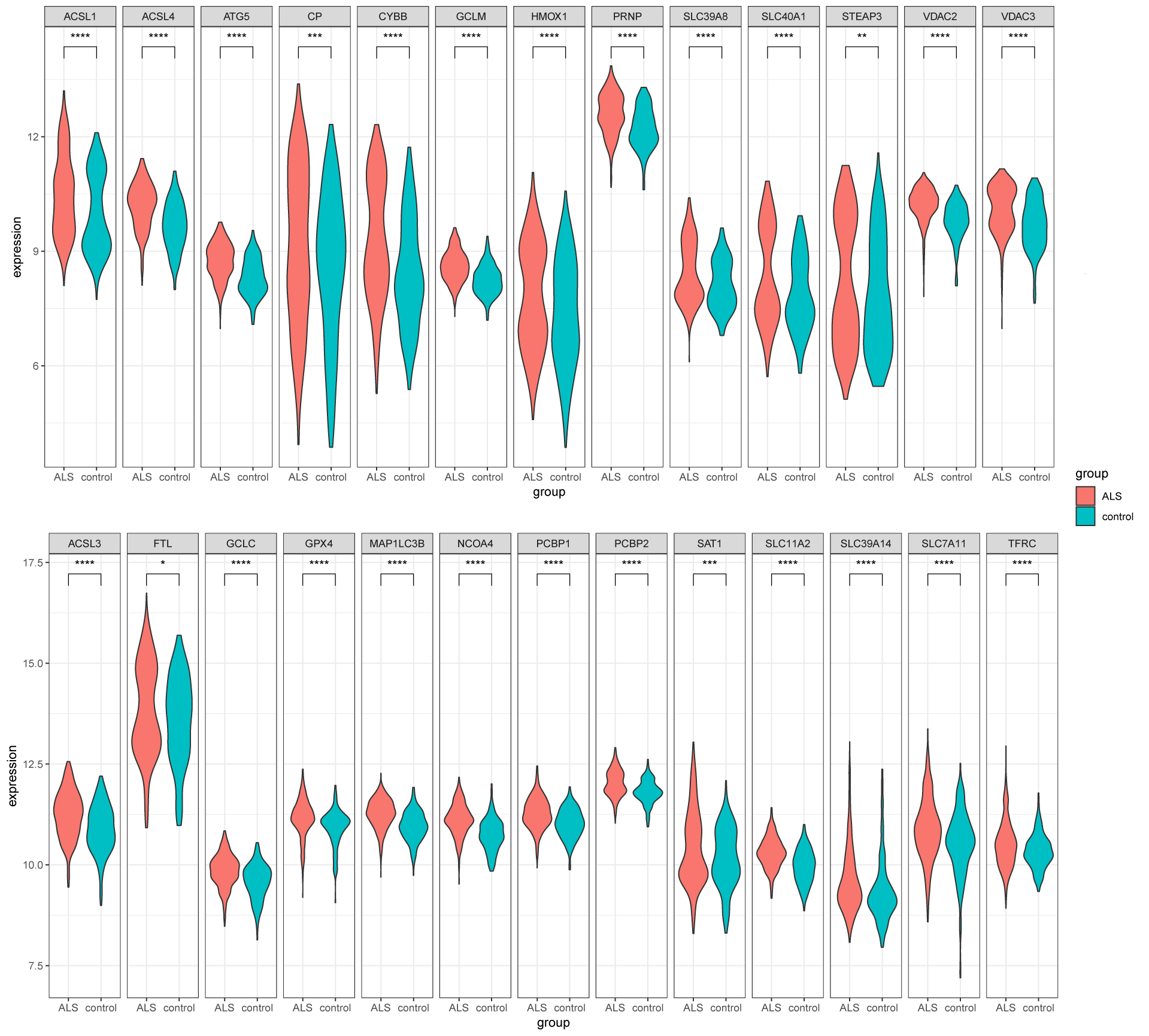

Supplement: Supplementary file 5 [file Image1.TIF]
