# Supplementary material for: The Classification and Prediction of Ferroptosis-Related Genes in ALS: A Pilot Study
Source: Front Genet. 2022 Jul 8;13:919188. doi: 10.3389/fgene.2022.919188 (PMC9305067; doi:10.3389/fgene.2022.919188)
Supplement: Supplementary file 7 [file Table1.DOC]

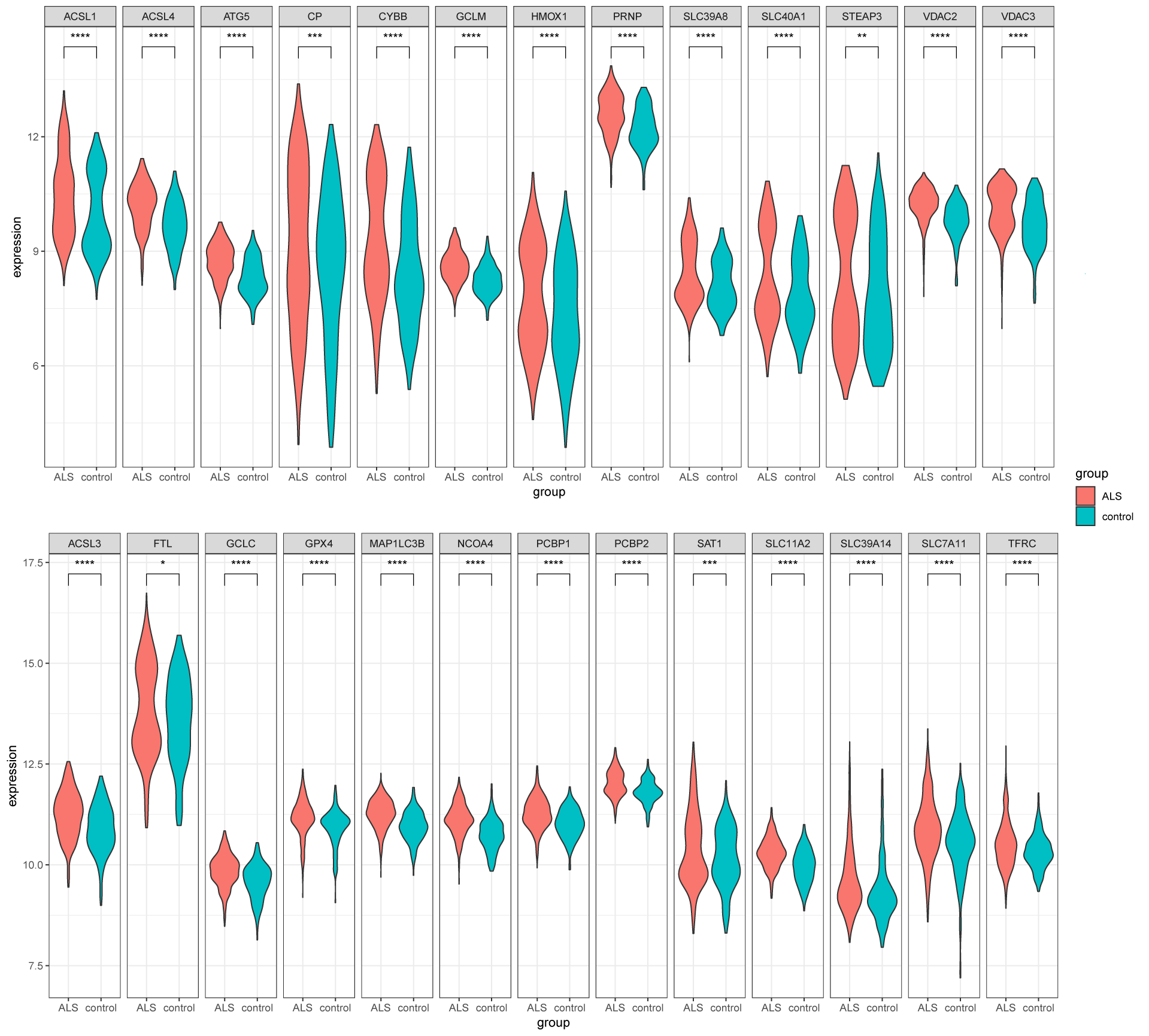


**Supplimentary 1** T-test of 26 genes in Ferroptosis pathway enriched by GSEA.

Note: Orange color represents ALS, turquoise color represents controls. *P < 0.05; **P < 0.01; ***P < 0.001; ****P < 0.0001.
