# Supplementary material for: The Classification and Prediction of Ferroptosis-Related Genes in ALS: A Pilot Study
Source: Front Genet. 2022 Jul 8;13:919188. doi: 10.3389/fgene.2022.919188 (PMC9305067; doi:10.3389/fgene.2022.919188)
Supplement: Supplementary file 8 [file Table2.DOC]

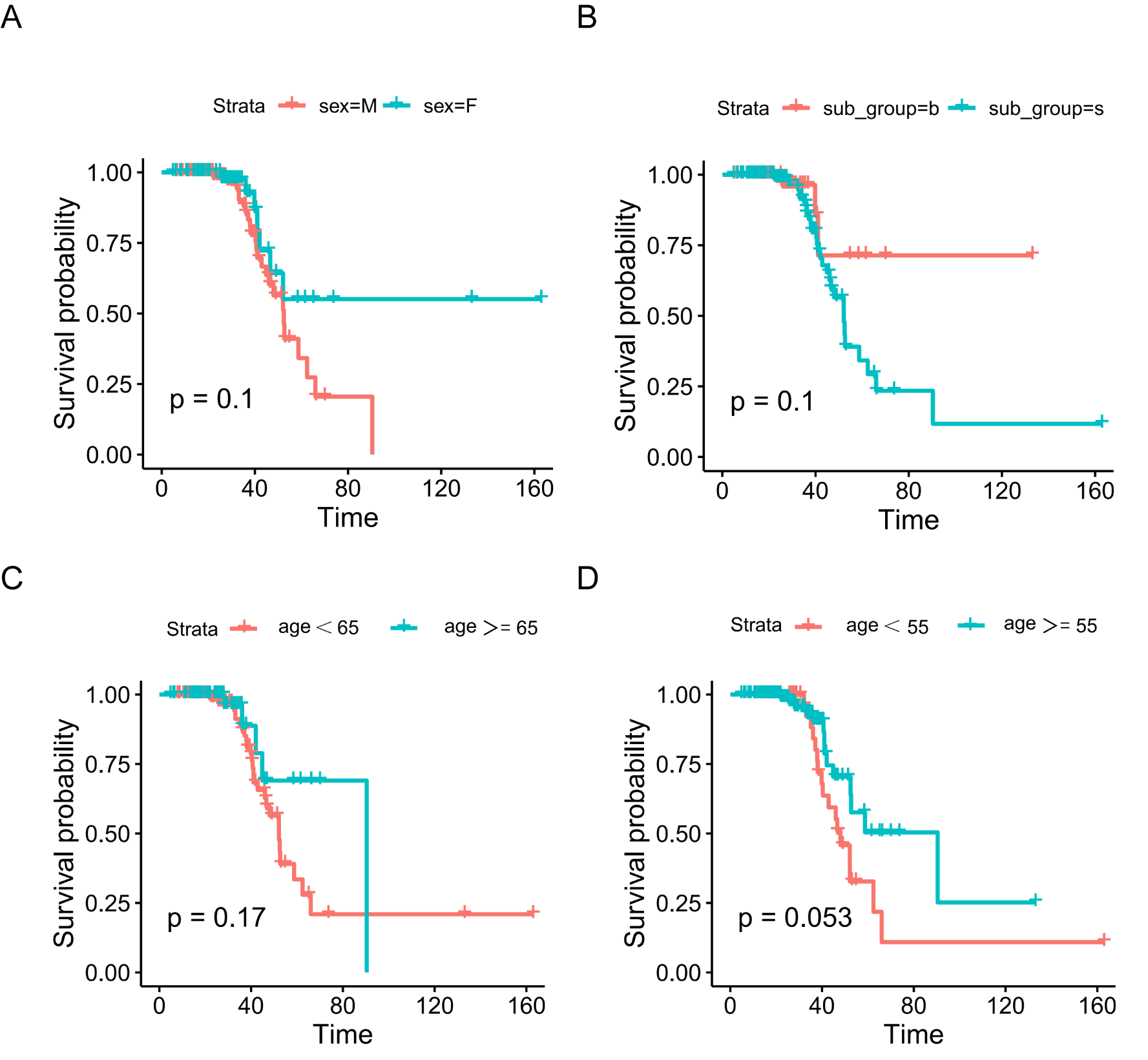


**Supplimentary 3** Kaplan-Meier survival curve of clinical features in ALS. (A) for male and female. (B) for spinal onset and bulbar onset of ALS. (C) and (D) showing Kaplan-Meier curves for different age nodes. ≥65 years  and ≥ 55 years, respectively. M, male; F, female; S, spinal; B, bulbar
